# A complete workflow for utilizing Monte Carlo toolkits in clinical cases for a double‐scattering proton therapy system

**DOI:** 10.1002/acm2.12473

**Published:** 2018-11-13

**Authors:** Leland Muller, Michael Prusator, Salahuddin Ahmad, Yong Chen

**Affiliations:** ^1^ Department of Radiation Oncology University of Oklahoma Health Sciences Center Oklahoma City OK USA

**Keywords:** Monte Carlo, proton, visualize

## Abstract

The methods described in this paper allow end users to utilize Monte Carlo (MC) toolkits for patient‐specific dose simulation and perform analysis and plan comparisons for double‐scattering proton therapy systems. The authors aim to fill two aspects of this process previously not explicitly published. The first one addresses the modeling of field‐specific components in simulation space. Patient‐specific compensator and aperture models are exported from treatment planning system and converted to STL format using a combination of software tools including Matlab and Autodesk's Netfabb. They are then loaded into the MC geometry for simulation purpose. The second details a method for easily visualizing and comparing simulated doses with the dose calculated from the treatment planning system. This system is established by utilizing the open source software 3D Slicer. The methodology was demonstrated with a two‐field proton treatment plan on the IROC lung phantom. Profiles and two‐dimensional (2D) dose planes through the target isocenter were analyzed using our in‐house software tools. This present workflow and set of codes can be easily adapted by other groups for their clinical practice.

## 1 INTRODUCTION

Monte Carlo techniques are important tools in radiotherapy due to their ability to accurately calculate dose in heterogeneous mediums.^1^, ^2^ This is particularly true in proton therapy where there are few commercially available algorithms capable of accurately handling the particle transport through heterogeneities and calculating secondary neutron dose.^3^ Monte Carlo methods therefore have widely been used for dose calculation in the field of medical physics. One of the Monte Carlo packages commonly used, particularly in proton therapy, is GEANT4, which has been packaged for proton therapy in the TOPAS platform.^2^

The Geant4 simulation toolkit, originally created for the study of high‐energy particles, currently has applications in many fields that include nuclear physics, astrophysics, high‐energy physics, aerospace, and of course medical physics.^4^ TOPAS wraps and extends the toolkit so as to provide an easily accessible tool for end users in the radiation oncology and medical physics fields to simulate proton doses. It operates by simulating particle transportation through a user‐defined geometry and calculates the radiation dose delivered. In double scatter proton therapy, protons travel along a beam line composed of components which are either machine specific or treatment field specific. For each treatment plan, there are typically 1–3 fields. Simulation of treatment fields thus needs these treatment field‐specific components to be modeled and brought into the beam geometry in a simple manner.

Our institution operates the MEVION S250 double‐scattering proton system.^5^, ^6^ With this treatment unit, each field has two unique components: a compensator and an aperture. The compensator is an acrylic object designed to conform the distal edge of each beam to the target while the aperture collimates the edges of the beam to the target. These components are designed in the treatment planning software. To calculate dose to a given patient or phantom, our treatment planning software, Eclipse ‐ Varian Medical Systems, Palo Alto, utilizes a pencil beam convolution algorithm.^7^ This algorithm has limitations in its accuracy in highly heterogeneous tissue such as lung.^8^ It is therefore often appropriate, for research and clinical purposes, to calculate the dose using an alternative method.^4^

In this paper, we present a methodology of using 3D Slicer^8^ in conjunction with MATLAB (The MathWorks, Inc., Natick, MA, USA) to bring field‐specific components generated by the treatment planning software into the simulation space. We then present in‐house codes developed to visualize and compare the calculated doses between the simulations and the actual treatment plan. Previous works have provided the guiding information of using the MC toolkits for creating general geometries for simulation purpose.^9^ However, the authors feel there are still gaps to be addressed in order to facilitate clinical practice. We believe that the present procedure will offer end users a seamless workflow and sets of tools for both clinical and research purposes.

## 2 MATERIALS AND METHODS

Two procedures were developed in the present study. The first provides a detailed workflow for the creation of 3D models for field‐specific components, which can be imported into the simulation geometry for double‐scattering systems. The second describes the steps of visualization and comparison of doses generated by MC toolkits and treatment planning system.

**Figure 1:**
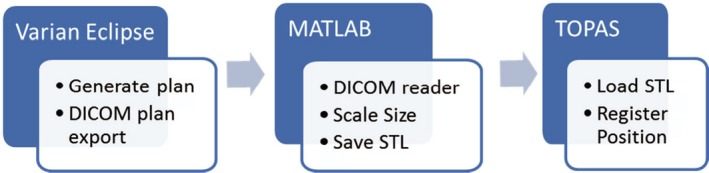
**Process for loading compensators into** **TOPAS** **.**

**Figure 2:**
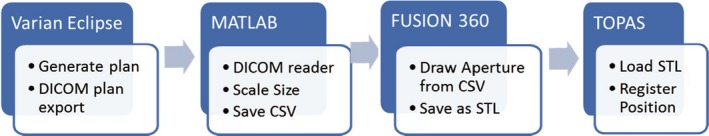
**Process for loading apertures into** **TOPAS** **.**

To start the simulation, we have first modeled our proton machine in TOPAS and benchmarked against measured commissioning beam data.^5^ Then the apertures and compensators for each of the treatment fields were designed with the treatment planning software, Varian Eclipse, and exported as part of the DICOM RN plan file. TOPAS and Geant4 have the built‐in capability to import these structures into simulation geometry once they are in stereolithographic (STL) format.^10^ There is therefore a need for a solution that converts each aperture and compensator file to this format. The method to convert from DICOM to STL is shown schematically in Figs. 1 and 2 for compensators and apertures, respectively. To address the gantry rotation in the simulation, we found it more straightforward to rotate the CT data set to match the angle of incidence of the proton beam. This was due to the restriction of TOPAS allowing phase space sources to be translated or rotated in the simulation space. Each of the fields was simulated separately with the corresponding patient‐specific components in place. The composite dose from the fields was calculated and displayed via summation of the outputs from two DICOM dose data sets.

### 2.A Compensator

The information necessary to define each compensator was expressed though several tags in the RN Plan file. Specifically, the size of the compensator was given by the corner position, number of rows, number of columns, and pixel dimensions, and the thickness was given as a dimensionless 2D array. The method, detailed in the Appendix A, uses this information to define a 3D array which is then converted into “solid” and then saved in STL format.

**Figure 3:**
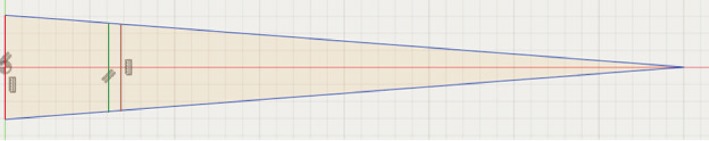
**An approximation of geometric projection of the field from the source (right side) to the nominal isocenter. The size of components is given by their projection at isocenter. The approximate position and sizes of the compensator and aperture are given at the green and brown lines respectively. When modeling these components, it is necessary to scale them to their position in the beam line.**

When inserting the device models into the beam line geometry, the size of the compensator needed to be scaled to their actual size determined through its position in the beam line as shown in Fig. 3. The dimensions of the compensator in DICOM format were given as projection at the isocenter of the treatment delivery system. To account for this, the size was geometrically scaled to the location of the component in the beam line based on the virtual source axis distance (VSAD) (300A, 030A), the “applicator” or snout position (300A, 030D), and the aperture thickness (300A, 0100), as shown in Eq. (1).

(1) $$MagFactor=\frac{VSAD-SnoutPosition+CompensatorThickness}{\mathrm{VSAD}}$$

### 2.B Aperture

Whereas the compensator is defined as an array of thicknesses, the aperture shape was given as a series of coordinates defining perimeter of the cutout region. These coordinates were reshaped and then scaled with Eq. (2) to its respective position in the beam line.

(2) $$MagFactor=\frac{VSAD-SnoutPosition}{\mathrm{VSAD}}$$

A grid was defined with the dimensions of the aperture and assigned values of 1 if the position in the grid is inside of a circle defining the outer edge of the aperture and outside of the coordinates defining the aperture. This grid was converted to an STL file with the same method as the compensator (Appendix B). Because there are sections of this shape with zero thickness, we clean up the edges of the aperture using the Autodesk software, Netfabb, which is free for academic uses. Additionally Netfabb was used to reduce the file size by manipulating the mesh to reduce the number of triangles with zero deformation. This model of the aperture is then saved and loaded into the MC simulation.

### 2.C Dose visualization and comparison

Both TOPAS (version 3.1.p2) and Geant4 (version 10.3) score the calculated dose values from each simulation onto a preset calculation grid. Geant4 saves the dose with a series of copyids, and TOPAS has the added functionality of saving the results as a DICOM RTDose file. In both cases additional software is required to display the dose and perform quantitative analysis. There are a number of tools which can be helpful for the purpose of evaluating and comparing doses.^11^ Among these are isodose lines, gamma analysis, DVH analysis, and plan normalization. In evaluating the dose to a given organ, DVH analysis is critical in assessing the likelihood of given biological endpoints. Additionally, comparing doses calculated using different programs and algorithms can be beneficial. For this, gamma analysis is a commonly used tool.^12^, ^13^ The open source software 3D Slicer with the radiotherapy extension provides these tools through the extension “Slicer RT.”

Doses generated in TOPAS and saved as DICOM RT Dose files need only to be normalized and have the position corrected before they were loaded directly into 3D Slicer.

The raw value for the simulated dose in each voxel corresponds with the total number of incident particle initiated for each run, while the values from a commercial treatment planning software are normalized to the prescription value. To be able to compare them side by side, we utilized the DICOM tag “Dose Grid Scaling”. The dose at a particular position is given by the value for the voxel in the dose grid multiplied by the dose grid scaling, so normalization was achieved through multiplying the “Dose Grid Scaling” tag by the ratio of the dose at isocenter in the TPS and MC. A median filter was also applied for the simulated dose grid to account for noise in the simulation dose before comparing to the treatment planning values.

Another useful tool in radiotherapy is acquiring dose profiles. This functionality is acquired through the simultaneous use of both 3D Slicer and Matlab. First the coordinates of the desired profile were found in 3D Slicer. Then these coordinates are then converted to voxel locations, and a profile was plotted in Matlab.

The output files from Geant4 simulations require an additional step to process. This step was done through converting the copyids into coordinates in a 4‐dimensional (4D) dose array (Appendix C). Additionally there are a number of tags in DICOM dose file which are necessary for other applications to properly identify it as a dose file.^14^ The tag which identifies the file as an RT Dose file is the “modality” tag (008,0060) given as “RTDOSE”. RT dose files share four tags with CTs: “Image Position” (0020,0032), “Rows” (0028, 0010), “Columns” (0028,0011) and “Pixel Spacing” (0028,0030). Unlike CT files, RT Dose files have all of the planes stored in a single file, so there are two additional tags for RT Dose files which define this geometry. These tags are “Number of Frames” (0028,0008) and “Grid Frame Offset Vector” (3004,000C). The “Number of Frames” is the number of slices in the z direction and the “Grid Frame Offset Vector” is a vector containing the distance from the “Image Position” in the z direction for each slice. The “series description” tag was updated to allow for easy identification of the correct file when loading it. These tags were defined for a given geometry and saved using the “dicomwrite” function with Matlab image processing toolbox version 2011b or later.

## 3 RESULTS

**Figure 4:**
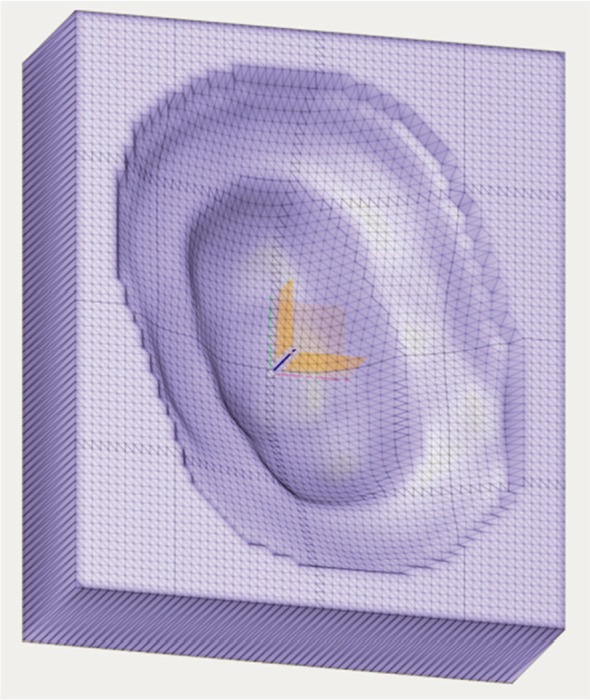
**An example compensator displayed in Fusion 360.**

**Figure 5:**
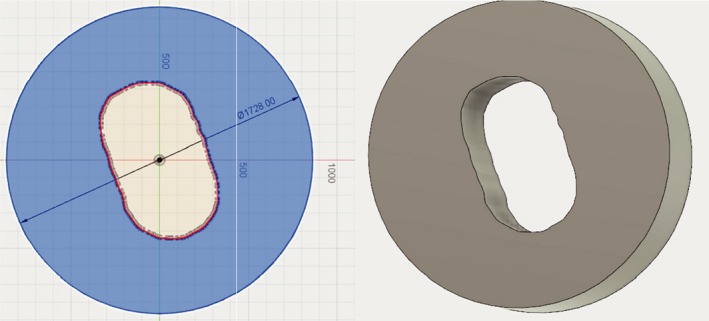
**Aperture sketch (left) and extruded 3D body in Fusion 360 (right). The diameter of aperture was set with the dimension tool.**

The procedure described above was performed on an example case using the IROC lung phantom.^15^ The phantom is composed of tissue, lung, bone, and heart equivalent material, and has a target in the center which moves during treatment delivery. To account for the motion a 4D CT scan was done with our CT Discovery using the Varian RPM system. A treatment was plan with two fields was made using the maximum intensity projection from the 4D scan to delineate the target. The compensators and apertures were converted to STL format and were shown with Netfabb in Figs. 4 and 5, respectively. All simulations were done on our Dell PowerEdge T420 computing server equipped with two Xeon E5‐2400 CPUs and 64 GB memory. For each field, 2 × 10^8^ initial proton particles were used and the computing time lasts from 2 to 3 h.

**Figure 6:**
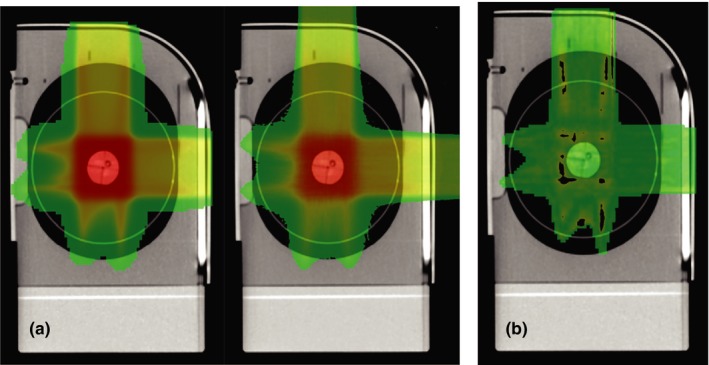
**Dose comparisons between Eclipse and** **TOPAS** **simulation. (a) a side by side comparison of Eclipse (Left) and** **TOPAS** **(Right) doses. (b) shows gamma analysis of the two calculations. Points with gamma values between 0 and 1 are displayed. These points are taken to be “passing”. Points without color either have a gamma index greater than 1 or are below the threshold of 10%**

**Figure 7:**
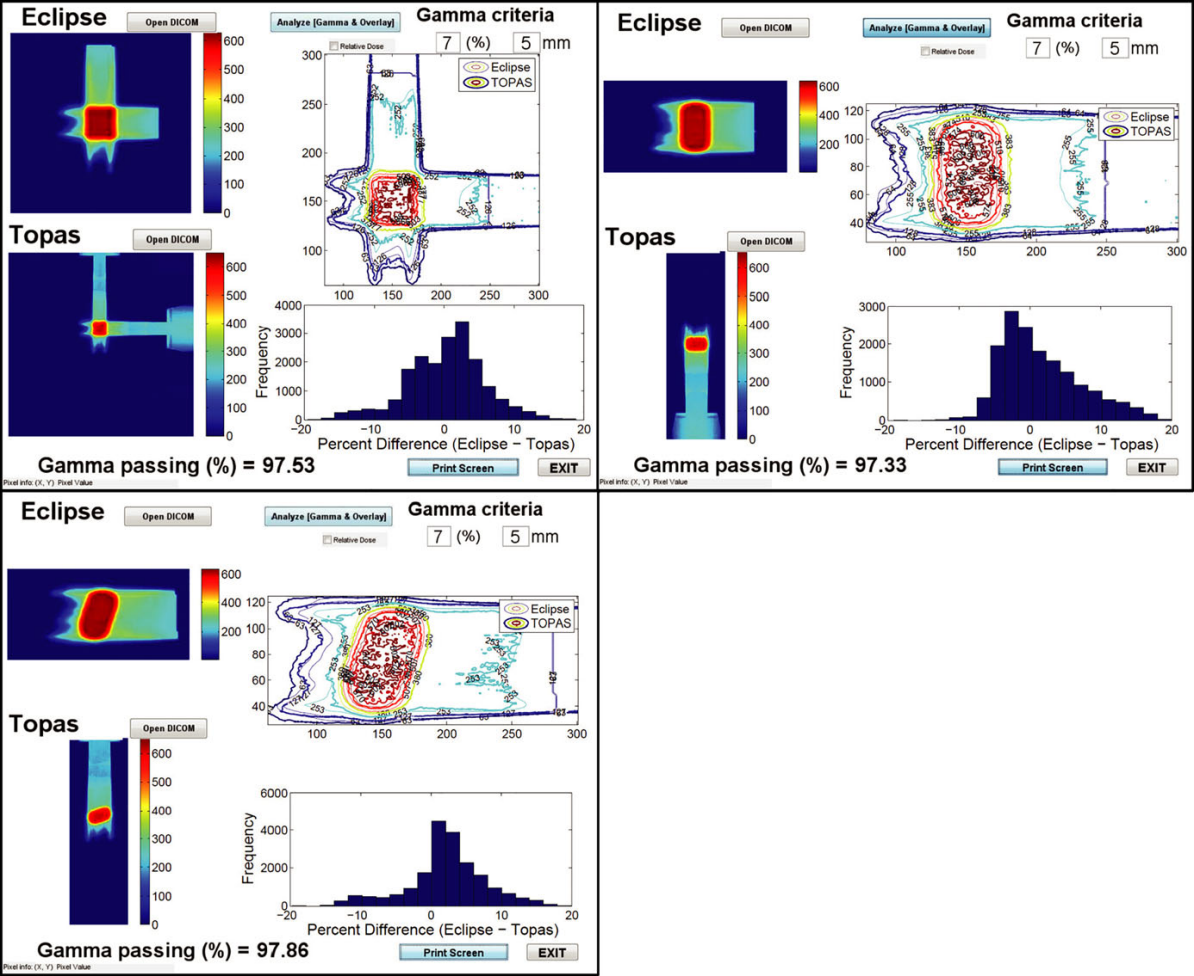
**Gamma Analysis for three planes through isocenter. axial (upper Left), coronal, (upper right) and sagittal (lower left). Color wash images of the dose from Eclipse and TOPAS are shown on the left side of each panel. Isodose lines from each dose source are superimposed with corrected orientations in the upper right of each panel. A histogram of the dose difference between Eclipse and TOPAS is shown in the bottom right. The histograms are truncated to only show dose deviations less than 20%. And the Eclipse dose is used as the reference dose for gamma analysis.**

Normalized‐ and filtered‐simulated doses and Eclipse‐calculated doses were then loaded into 3D Slicer. The dose from each was shown side by side in Fig. 6(a). A three‐dimensional (3D) gamma analysis was performed using criteria of 5 mm/7% with a 10% threshold and the gamma passing rate was found to be 90.68%. The passing points (points with a gamma index value between zero and 1) were shown in Fig. 6(b). Two‐dimensional gamma analyses were also performed in planes (axial, coronal, and sagittal) through isocenter using an in house Matlab GUI. The gamma passing rates were tabulated in Table 1 and the overlaid isodose lines and dose difference histograms were shown in Fig. 7.

**Table 1:** Gamma analysis of the film measurements vs calculated doses as well as the between TOPAS and Eclipse. Comparisons with film were done by IROC and the comparisons between TOPAS and Eclipse were done at the University of Oklahoma

Plane	ECLIPSE vs film (%)	TOPAS vs film (%)	TOPAS vs eclipse (%)
Axial	94	99	98
Coronal	94	99	97
Sagital	92	99	98
Average	93	99	98

**Figure 8:**
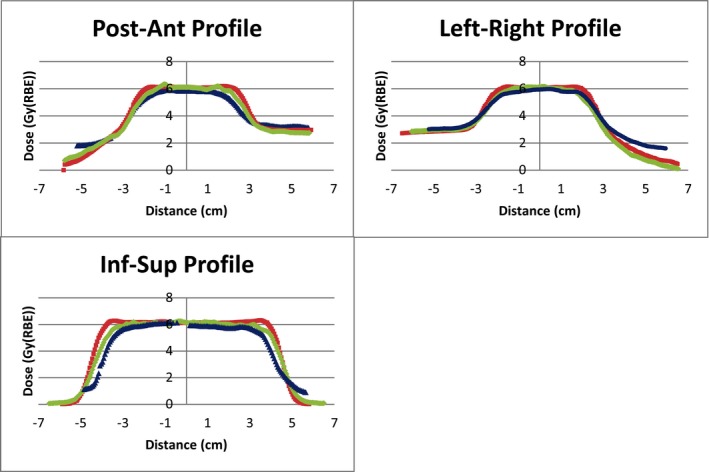
Profiles through the target in each orthogonal direction. The film, TOPAS, and Eclipse doses are shown with blue triangles, green diamonds, and red squares, respectively. In each profile there is a better agreement between the film and TOPAS than between the film and Eclipse calculations. The film was processed by IROC.

The TOPAS‐simulated doses and Eclipse‐calculated doses were compared with the film placed through each plane of the target in the IROC phantom. Gamma analysis of the films was performed by IROC with criteria of 7% and 5 mm. IROC uses this criteria to account for the effects of film quenching.^15^, ^16^ Results from the gamma analysis were shown in Figs. 6 and 7. Profiles from the film, Eclipse, and TOPAS were shown in Fig. 8. In this particular case, we observe acceptable agreement between Eclipse doses and film measurements as per IROC standards of ≥80% gamma passing rate. TOPAS agreement with the film was better than that for Eclipse as is expected in a highly heterogeneous phantom. In both cases, the accuracy of the dose calculation is affected by the fact that target motion during dose delivery is not accounted for. This limitation of the modeling gives a better agreement between the TPS and TOPAS than between either and the film.

## 4 CONCLUSION

In this paper we detail procedures for modeling treatment field‐specific components and performing MC simulation for double‐scattering proton therapy system previously unpublished. This procedure also allows for users to easily evaluate and compare the doses between commercial treatment planning software and MC simulations. All Matlab code used in this publication is available freely so other in the community can use these methods. It can be found at https://www.mathworks.com/matlabcentral/fileexchange/68660-toolkits-for-monte-carlo-dose-simulation-and-visualization

## CONFLICT OF INTEREST

The authors certify that they have no affiliations with or involvement in any organization or entity with any financial interest or nonfinancial interest in the subject matter or materials discussed in this manuscript.

## APPENDIX A

### A.1

The following appendix provides the main details and explanations about data manipulation performed in Matlab. The full code is available on the Mathworks website at: https://www.mathworks.com/matlabcentral/fileexchange/68660-toolkits-for-monte-carlo-dose-simulation-and-visualization.

### Compensator

For a given field (i) in a DICOM plan “info”, the DICOM tags for compensator are as follows:

$$\begin{array}{cc} & \text{Position}\;=\text{(300A,00EA) = info.IonBeamSequence.Item}\_\text{(i).}\\ & \;\text{IonRangeCompensatorSequence.Item}\_\text{1.CompensatorPositiom}\\ & \text{Rows}\;=\text{(300A,00E7) = info.IonBeamSequence.Item}\_\text{(i).}\\ & \;\text{IonRangeCompensatorSequence.Item}\_\text{1.CompensatorRows}\\ & \text{Columns}=\text{(300A,00E8) = info.IonBeamSequence.Item}\_\text{(i).}\\ & \;\text{IonRangeCompensatorSequence.Item}\_\text{1.CompensatorColumns}\\ & \text{PixelSize}=\text{(300A,00E9) = info.IonBeamSequence.Item}\_\text{(i).}\\ & \;\text{IonRangeCompensatorSequence.Item}\_\text{1.CompensatorPixelSpacing}\\ & \text{Thickness = (300A,00EC) = info.IonBeamSequence.Item}\_\text{(i)}.\\ & \;\text{IonRangeCompensatorSequence.Item}\_\text{1.CompensatorThicknessData} \end{array}$$

The first step in creating a model of the compensator is to create a properly scaled plane perpendicular to the beamline. This plane is defined by x and y vectors composed of evenly spaced points, centered at zero, where the number of points in the x and y vectors is given by the number of rows and columns, respectively. A border is applied to provide full scatter conditions. 
border = 5
x=(-Comp_pixel*border):Comp_pixel:((Comp_c{i})*Comp_pixel
+Comp_pixel*border-0.1);
x=x-(Comp_c{i}-1)/2*Comp_pixel;
y=-((Comp_r{i})*Comp_pixel+Comp_pixel*border):Comp_pixel:
(Comp_pixel*border-0.1);
y=y-(Comp_r{i}-1)/2*Comp_pixel;

The x and y vectors are then scaled and converted to a coordinate matrix with the Matlab “meshgrid” function. 
x=x*mag_factor(i);
y=fliplr(y*mag_factor(i));
[x,y] = (meshgrid(double(x),double(y)));

Now that we have the scaled grid of the compensator.

The thickness of the compensator is reformatted for the number of rows and columns representing it and the border is applied to the edges. The thickness of the border is the maximum thickness of the compensator.
 dim1 = raw(i).*ones(size(thickness,1),border);
 thickness = [dim1,thickness,dim1];
 dim2 = raw(i).*ones(border,size(thickness,2));
 thickness = [dim2;thickness;dim2];

The surface defined by the x, y and thickness variables is used to define the solid shape using triangle faces and vertices. This solid shape is in turn saved in STL format
[fv] = surf2solid(x,y,thickness,’ELEVATION’,0);
 stlwrite((strcat(‘RC_’,num2str(i),’_Matlab’,’.stl’)),fv)

## APPENDIX B

### B.1

The following appendix provides the main details and explanations about data manipulation performed in Matlab. The full code will be made available on the Mathworks website.

### Aperture

The aperture is defined by its thickness, diameter, and area to be cut out. The DICOM tags for this information are given as follows for a plan named “info”. 
Thickness = (300A,0100)= info.IonBeamSequence.Item_1.IonBlockSequence.Item_1.BlockThickness
NumPoints = (300A,0104)= info.IonBeamSequence.Item_1.IonBlockSequence.Item_1.BlockNumberOfPoints
Data = (300A,0106)= info.IonBeamSequence.Item_1.IonBlockSequence.Item_1.BlockData

The data is then converted into coordinate pairs according the number of points and scaled to their position in the beam line, 
mag_factor_B(i)=(VSAD(i,1)-BlockPosit(i))/VSAD(i,1);
 Block{i} = reshape(Block{i},2,NumPoints(i))’*mag_factor_B(i);

These points are then mapped onto a matrix. All points in that matrix are assigned a value of 1 if they lie outside of the block coordinates, and inside a circle the size of the aperture. This is performed through the lines below. The diameter variable is set to match the diameter of the aperture. 
Mesh = ones(ceil(Diameter/Resolution));
InCircle = zeros(ceil(Diameter/Resolution));
coordinates = zeros(size(Mesh,1)*size(Mesh,2),2);
num = 1;
for x = 1:size(Mesh,1)
for y = 1:size(Mesh,2)
coordinates(num,:) = [x*Resolution,y*Resolution];
 num = num+1;
 if (x*Resolution-Diameter/2)^2+(y*Resolution-
Diameter/2)^2<=(Diameter/2)^2
 InCircle(x,y) = 1;
 else
 continue
 end
 end 
end
X=(coordinates(:,1)-Diameter/2);
Y=(coordinates(:,2)-Diameter/2);
out = ˜inpolygon(X,Y,Block{i}(:,1),Block{i}(:,2));
out = reshape(out,size(Mesh,1),size(Mesh,2));
AP = InCircle.*out*60;
[x,y] = (meshgrid((1:size(Mesh,1))*Resolution,(1:size(Mesh,2))*Resolution));

The surface defined by the x, y, and AP variables is used to define the solid shape using triangle faces and vertices. This solid shape is in turn saved in STL format
 [fv] = surf2solid(x,y,AP,’ELEVATION’,0);
 stlwrite((strcat(‘AP_’,num2str(i),’_Matlab’,’.stl’)),fv)

The produced STL file will have points defined across the entire square of the meshgrid. This points need to be cleaned up before loading the aperture into the MC simulation. We found that the easiest way to do this is with the Autodesk program Netfabb, which is free for academic purposes. This model of the aperture can now be saved and loaded into the MC simulation. The aperture file size can be reduced by manipulating the mesh to reduce the number of triangles used to define the object shape.

## APPENDIX C

### C.1

The conversion from CopyID to a DICOM format dose grid is given below.

$$z=floor\left(\frac{CopyID(i)}{NX*NY}\right)$$

$$y=floor\left(\frac{CopyID(i)-z*NX*NY}{\mathrm{NX}}\right)$$

x=(copyID(i)−z∗NX∗NY−y∗NY)

dose(iy+1,ix+1,1,iz+1) = Dose(i);
dicomwrite(dose,’file name’,tag information,’CreateMode’,’copy’);

NX=numberofcolumns,NY−numberofrows,NZ=numberofslicess
